# Molecular mechanism for strengthening E-cadherin adhesion using a monoclonal antibody

**DOI:** 10.1073/pnas.2204473119

**Published:** 2022-08-03

**Authors:** Bin Xie, Allison Maker, Andrew V. Priest, David M. Dranow, Jenny N. Phan, Thomas E. Edwards, Bart L. Staker, Peter J. Myler, Barry M. Gumbiner, Sanjeevi Sivasankar

**Affiliations:** ^a^Biophysics Graduate Group, University of California, Davis, CA, 95616;; ^b^Department of Biomedical Engineering, University of California, Davis, CA, 95616;; ^c^Seattle Children’s Research Institute, Center for Developmental Biology and Regenerative Medicine, Seattle, WA, 98101;; ^d^Department of Biochemistry, University of Washington, Seattle, WA, 98195;; ^e^Seattle Structural Genomics Center for Infectious Disease, Seattle, WA, 98109;; ^f^UCB Pharma, Bainbridge Island, WA, 98110;; ^g^Center for Global Infectious Disease Research, Seattle Children’s Research Institute, Seattle, WA, 98109;; ^h^Department of Pediatrics, University of Washington, Seattle, WA, 98195

**Keywords:** E-cadherin, strand–swap dimer, antibody, 19A11, adhesion

## Abstract

E-cadherin (Ecad) is an essential cell–cell adhesion protein with tumor suppression properties. The adhesive state of Ecad can be modified by the monoclonal antibody 19A11, which has potential applications in reducing cancer metastasis. Using X-ray crystallography, we determine the structure of 19A11 Fab bound to Ecad and show that the antibody binds to the first extracellular domain of Ecad near its primary adhesive motif: the strand–swap dimer interface. Molecular dynamics simulations and single-molecule atomic force microscopy demonstrate that 19A11 interacts with Ecad in two distinct modes: one that strengthens the strand–swap dimer and one that does not alter adhesion. We show that adhesion is strengthened by the formation of a salt bridge between 19A11 and Ecad, which in turn stabilizes the swapped β-strand and its complementary binding pocket. Our results identify mechanistic principles for engineering antibodies to enhance Ecad adhesion.

## Significance

E-cadherin is an essential cell–cell adhesion protein that plays key roles in the formation and maintenance of epithelial tissue. Deficiencies in E-cadherin adhesion are associated with the metastasis of a number of highly invasive cancers. Recently, monoclonal antibodies with potential therapeutic applications have been developed that strengthen E-cadherin adhesion. Here, we resolve the molecular mechanisms that underlie the function of these antibodies. We show that the monoclonal antibody 19A11 binds to E-cadherin near its primary adhesive motif and strengthens adhesion by forming a key salt bridge that stabilizes the E-cadherin binding site. Our results identify mechanistic principles for engineering antibodies to enhance cadherin adhesion.

E-cadherin (Ecad) is an essential cell–cell adhesion protein that plays key roles in the formation of epithelial tissues and in the maintenance of tissue integrity. Adhesion is mediated by the *trans* binding of Ecad ectodomains (extracellular regions) from opposing cell surfaces. Deficiencies in Ecad adhesion result in the loss of contact inhibition and increased cell mobility (1) and are associated with the metastasis of gastric cancer (2), breast cancer (3), colorectal cancer (4), and lung cancer (5). Consequently, strategies that activate or strengthen Ecad adhesion may have potential applications in reducing cancer metastasis.

A powerful therapeutic approach that has been successfully used in regulating the binding of cell adhesion proteins are monoclonal antibodies (mAbs). For example, mAbs targeted against integrin adhesion proteins are used in the treatment of Crohn’s disease (6–8). Similarly, we have identified activating mAbs that target Ecad ectodomains and enhance cell–cell adhesion (9). In mouse models, one of these mAbs, 19A11, prevents the metastatic invasion of mouse lung cancer cells expressing human Ecad (10, 11). In addition, we have shown that 19A11 can enhance the Ecad epithelial barrier function and limit the progression of inflammatory bowel disease (12). Here, we resolve the molecular mechanisms by which mAb 19A11 strengthens Ecad adhesion.

We demonstrate that 19A11 strengthens adhesion by stabilizing strand–swap dimers, which are the predominant Ecad *trans* binding conformation. Strand–swap dimers are formed by the exchange of N-terminal β-strands (residues 1–12) between the outermost domains (EC1) of opposing Ecads. The exchange of β-strands results in the symmetric docking of a conserved anchor residue, tryptophan at the position 2 (W2), into a complementary pocket on the partner Ecad (13–15). Previous studies show that the two key structural and energetic determinants of Ecad strand–swap dimer formation are the stability of swapped β-strands (16) and their corresponding hydrophobic binding pockets (17). Using X-ray crystallography, molecular dynamics (MD) simulations, steered MD (SMD) simulations, and single-molecule atomic force microscopy (AFM), we show that 19A11 binding stabilizes both the β-strand and the hydrophobic pocket by forming key salt bridges. Our results identify the mechanistic principles underlying the activation of cadherin adhesion by mAbs.

## Results

### Crystal structure of 19A11 bound to Ecad.

**Fig. 1.**
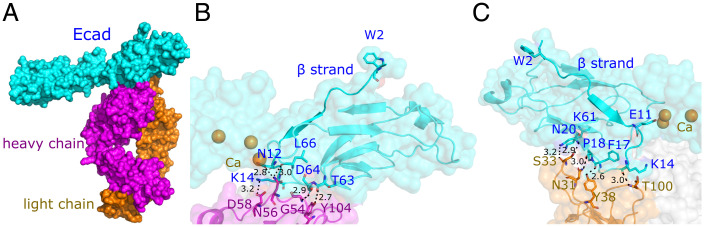
Structure of 19A11 bound to Ecad. (*A*) X-ray crystal structure of 19A11 Fab heavy chain (magenta) and light chain (orange) bound to Ecad EC1–EC2 domains (cyan). (*B*) Detailed view of the hydrogen bonds and salt bridges between 19A11 Fab heavy chain (magenta) and Ecad EC1 domain (cyan). (*C*) Detailed view of the interactions between 19A11 Fab light chain (orange) and Ecad EC1 domain (cyan). The distances between interacting atoms in (*B* and *C*) are shown in Å (black dashed lines).

We cocrystallized the EC1-2 domains of human Ecad and 19A11 antibody fragment (Fab) and determined the structure at a 2.2Å resolution (Fig. 1*A*; PDB accession code 6CXY). The structure refinement parameters are summarized in *SI Appendix*, Table 1. The crystal structure reveals that 19A11 recognizes two regions on the Ecad EC1 domain: residues 13 to 20 and residues 61 to 70. The binding of 19A11 does not cause gross conformational changes on Ecad, and the root mean square deviation (RMSD) between the protein backbone of the crystal structures in the presence and absence (PDB accession code 2O72) of 19A11 is only 0.3Å. A closer look at the Ecad binding interface shows that the binding of the antibody heavy chain and Ecad is primarily mediated by a salt bridge between K14 on Ecad and D58 on 19A11 (Fig. 1*B*) and four Ecad:mAb hydrogen bonds (N12:N56, side chain to side chain; T63:Y104, backbone to side chain; D64:G54, side chain to backbone; and L66:N56, backbone to side chain; Fig. 1*B*). In addition, the crystal structure shows five hydrogen bonds between Ecad and the 19A11 light chain (K14:T100, backbone to backbone; F17:Y38, backbone to side chain; P18:N31, backbone to side chain; N20:S33, backbone to side chain; and K61:S33, side chain to backbone; Fig. 1*C*). The strong interaction between 19A11 and residues 13 to 20 of Ecad located near the base of the N-terminal β-strand led us to hypothesize that these interactions may strengthen Ecad strand–swap dimers.

### Molecular mechanisms of 19A11-mediated stabilization of strand–swap dimers.

**Fig. 2.**
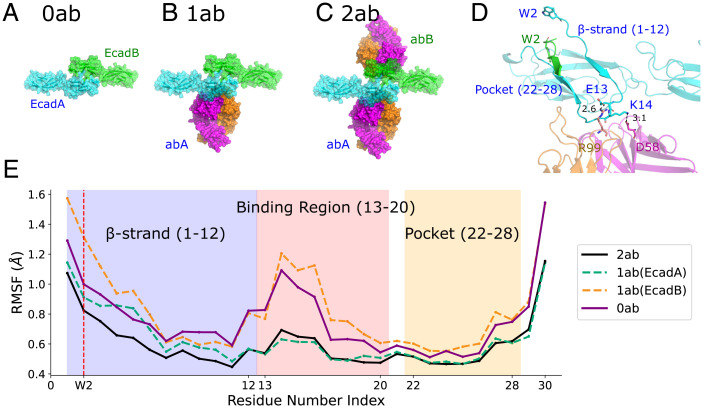
Binding of 19A11 stabilizes both the Ecad β-strand and the W2 hydrophobic pocket. MD simulations were performed with (*A*) the Ecad strand–swap dimer (EcadA and EcadB) in the absence of 19A11 (0ab), (*B*) the Ecad strand–swap dimer with a single 19A11 Fab (abA) bound to EcadA (1ab), and (*C*) the Ecad strand–swap dimer with two 19A11 Fabs (abA and abB) bound to both Ecads (2ab). (*D*) Ecad–antibody binding interface. Two salt bridges are observed: E13–R99 and K14–D58. The 19A11 binding region is located between the β-strand and the W2 hydrophobic pocket (referred to as “Pocket”) on Ecad. (*E*) Average RMSF values for residues 1–30 of Ecad in the 2ab case (solid black), EcadA in the 1ab case (dashed green), EcadB in the 1ab case (dashed orange), and Ecad in the 0ab case (solid purple). The W2 position is highlighted using a vertical dashed red line. The lower RMSF values show that the binding of 19A11 stabilizes the β-strand and the W2 hydrophobic pocket of both Ecads in the 2ab case while it only stabilizes EcadA (which is bound to 19A11) in the 1ab case.

To resolve the detailed molecular interactions between 19A11 and human Ecad, we performed MD simulations on three different structures: 1) Ecad strand–swap dimer (*EcadA* and *EcadB*) without 19A11 Fab (**0ab**, Fig. 2*A*; PDB accession code 2O72; species: human), 2) Ecad strand–swap dimer bound to a single 19A11 Fab (*abA* bound to *EcadA*) (**1ab**, Fig. 2*B*; PDB accession code 6CXY; species: human), and 3) Ecad strand–swap dimer bound to two 19A11 Fabs (*abA* bound to *EcadA* and *abB* bound to *EcadB*) (**2ab**, Fig. 2*C*; PDB accession code 6CXY). In the 0ab, 1ab, and 2ab conditions, we performed five independent simulations. Every MD simulation was performed for 60 ns, which was long enough for the RMSD relative to the structures at the start of the simulations to stabilize (*SI Appendix*, Fig. S1).

Since the β-strand and its complementary binding pocket are essential components of Ecad strand–swap dimers (Fig. 2*D*), we first tested whether there was a change in the stability of either region upon 19A11 binding. We tested the stability by measuring the root mean square fluctuations (RMSF, the SD of the atomic positions) of the corresponding α-carbon residues during the last 10 ns of all MD simulations (Fig. 2*E*). The average RMSFs for EcadA and EcadB in the 1ab conditions were calculated separately since only EcadA is bound to an antibody (Fig. 2*E*, dashed green and dashed orange lines). The average RMSF for Ecads in the presence and absence of the antibody showed that 19A11 binding reduces the rmsf of its corresponding binding regions on Ecad (residues 13–20). The binding of 19A11 also stabilizes the two adjoining regions, the β-strand (residues 1–12) and the partial complementary pocket (residues 22–28), which are essential for the strand–swap dimer formation.

**Fig. 3.**
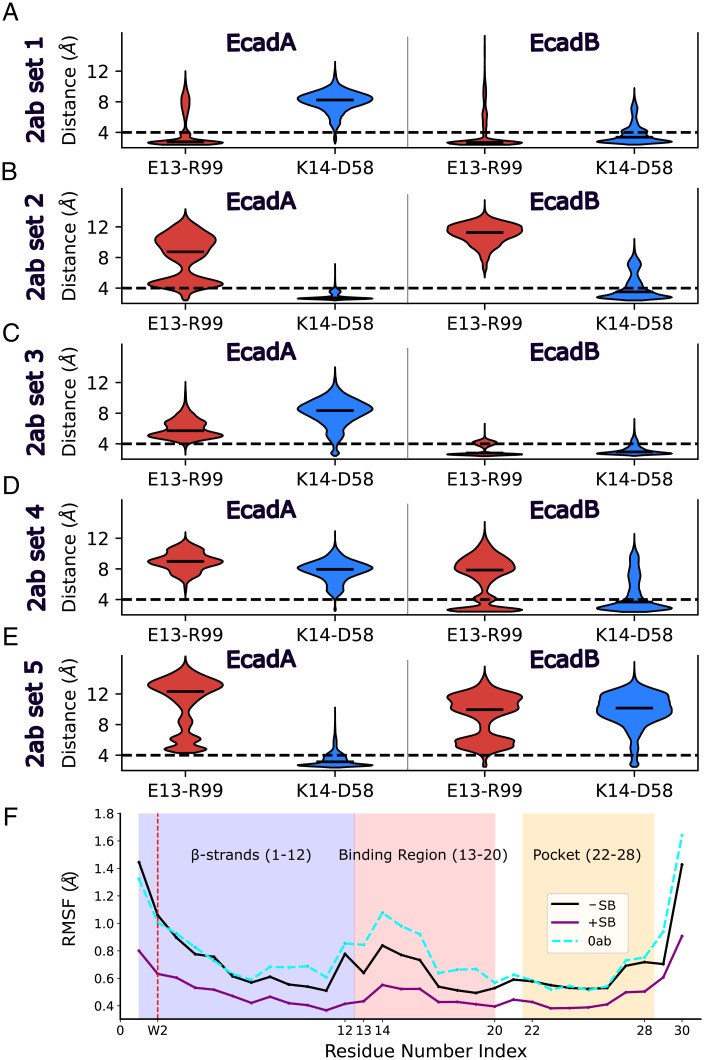
Salt bridges between 19A11 and Ecad stabilize the β-strand and the W2 hydrophobic pocket. (*A–E*) Violin plots of the distances between charged atoms in the E13–R99 and K14–D58 salt bridges measured during the last 40 ns of each 2ab MD simulation. The median distance is shown as a black line on each violin. Distances for EcadA and EcadB are shown in the left and right panels, respectively. Distances measured for E13–R99 interactions during the MD simulations are shown in red and charged atoms distances for K14-D58 are shown in blue. (*A*) simulation 1 (set 1), (*B*) simulation 2 (set 2), (*C*) simulation 3 (set 3), (*D*) simulation 4 (set 4), (*E*) simulation 5 (set 5). Both EcadA and EcadB form at least one salt bridge with the bound 19A11 in set 1 and set 2. However, only one of the Ecads formed a salt bridge with 19A11 in sets 3–5 (EcadB in set 3, EcadB in set 4, and EcadA in set 5). (*F*) Comparison of the average backbone RMSF values when an Ecad forms at least one salt bridge with its corresponding 19A11 (purple solid line), when an Ecad does not form at least one salt bridge with its corresponding 19A11 (black solid line), and in the absence of 19A11, i.e., 0ab (dashed cyan line). The RMSF for W2 is highlighted using a vertical dashed red line. The β-strand and the W2 hydrophobic pocket have a lower RMSF when a salt bridge is formed as compared to when no salt bridges are formed.

Next, we examined specific interactions between 19A11 and Ecad to determine the molecular mechanisms by which mAb binding stabilizes the β-strand and pocket region. We focused on two salt bridges that form between 19A11 and Ecad, downstream of the β-strand (Fig. 2*D*). The first salt bridge occurs between E13 on Ecad and R99 on the 19A11 light chain, while the second salt bridge forms between K14 on Ecad and D58 on the 19A11 heavy chain (Fig. 2*D*). We measured the distances between the salt bridges in every MD simulation, in both the 2ab (Fig. 3 *A–E*) and 1ab (*SI Appendix*, Fig. S2) conditions, from 20 ns to 60 ns. Based on the criterion that salt bridges form when the median distance between charged atoms is less than 4Å, we concluded that in 40% of the 2ab simulations (sets 1 and 2, Fig. 3 *A* and *B*), both Ecads form at least one salt bridge with the bound 19A11. However, in the remaining 60% of the simulations, only one of the Ecads formed at least one salt bridge with 19A11 (EcadB of set 3, EcadB of set 4, and EcadA of set 5; Fig. 3 *C–E*). Notably, the salt bridges formed by sets 3 to 5 in the 2ab simulations were similar to the 1ab condition (*SI Appendix*, Fig. S2), in that only one Ecad formed a salt bridge (since there was only one 19A11 Fab present in the 1ab condition).

When an Ecad formed at least one salt bridge with its corresponding 19A11, the average RMSF of the β-strand and pocket region was lower (Fig. 3*F*, purple line), demonstrating that salt bridge formation stabilizes both the β-strand and pocket region. In contrast, the stability of the β-strand and pocket region when an Ecad did not form a salt bridge (Fig. 3*F*, black line) was approximately the same as the 0ab condition (Fig. 3*F*, dashed cyan line). Based on these results, we concluded that 19A11 can interact with Ecad in two different modes: one that stabilizes the β-strand and the pocket region by forming either E13–R99 or K14–D58 interactions, and a second mode that does not stabilize the β-strand and the pocket region because the salt bridges are not formed.

### 19A11-mediated stabilization of strand–swap dimer leads to stronger Ecad adhesion.

**Fig. 4.**
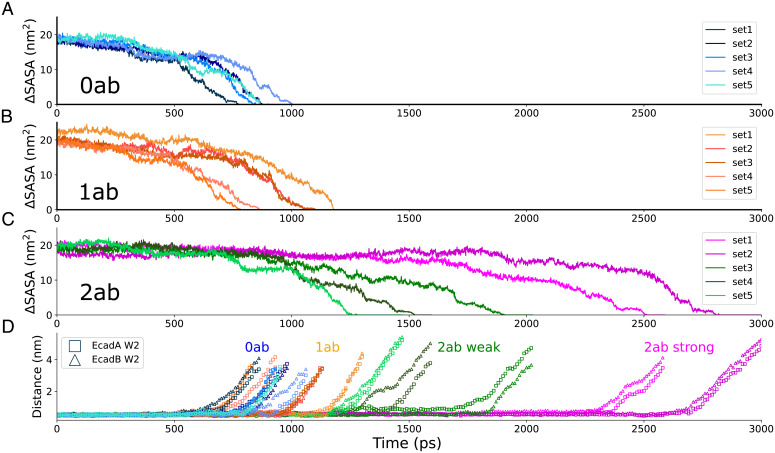
Adhesion strengthening requires two bound 19A11 antibodies to form salt bridges with partner Ecads. Constant-force SMD simulations with change in Ecad–Ecad interfacial area calculated from the ΔSASA, in the (*A*) 0ab condition, (*B*) the 1ab conditions, and (*C*) the 2ab conditions. (*D*) Distance between center of mass of W2 and the center of mass of the hydrophobic pockets in each of the constant-force SMD simulations. While the lifetimes of the Ecad–Ecad bonds are similar in the 0ab and 1ab and sets 3–5 of the 2ab condition, the lifetime of the Ecad–Ecad bond in sets 1–2 of the 2ab condition, where both interacting Ecads form at least one salt bridge with 19A11, are substantially longer (also see Movie S1).

Since each pocket region forms hydrogen bonds with a β-strand on its partner Ecad (17), we hypothesized that stabilizing the pocket and β-strand strengthens adhesion by retaining each β-strand in its swapped position and keeping each W2 inserted into its opposing pocket. To test this hypothesis, we performed SMD simulations. We fixed the C-terminal end of one Ecad in the final structure of each MD simulation and pulled on the other Ecad C terminus with a constant force of ∼665 pN (Movie S1). During each SMD simulation, we measured the interfacial binding area between the two Ecads, which we estimated using the change in the solvent-accessible surface area (ΔSASA) (18); a decrease of ΔSASA to zero corresponded to the rupture of the interacting *trans* dimer. In the 0ab condition (Fig. 4*A*), the ΔSASA dropped to zero at ∼900 ps while in the 1ab condition (Fig. 4*B*) the interactions between Ecads lasted marginally longer and broke at ∼1,000 ps. In contrast to the 0ab and 1ab conditions, we measured two populations in the 2ab condition: one that remained bound for a longer time and another that unbound on a timescale closer to the 0ab and 1ab conditions. Ecads in sets 1 and 2 of the 2ab condition interacted for ∼2,700 ps, suggesting a strong bound state, while in sets 3 to 5 the interactions only lasted for ∼1,600 ps (Fig. 4*C*).

As an additional measure of strand–swap dimer stability, we calculated the distances between the center of mass of W2 and the center of mass of its complementary binding pocket during the constant force SMD (Fig. 4*D*). These measurements show how long W2 is retained in the hydrophobic pocket because the β-strands remain in a swapped position. Similar to the ΔSASA measurement, W2 in the 0ab condition, 1ab condition, and sets 3 to 5 of the 2ab condition exited the binding pocket much earlier than 2ab sets 1 and 2 (Fig. 4*D*).

The salt bridges that were formed during the MD simulations were mostly retained in the SMD runs (*SI Appendix*, Fig. S3). Specifically, sets 1 and 2 of the 2ab conditions continued to retain at least one salt bridge with each Ecad, while only one Ecad in sets 3, 4, and 5 formed a salt bridge. No additional salt bridges between Ecad and the antibody were formed during the SMD simulations. Furthermore, hydrogen bonds and electrostatic interactions between the two Ecads, in the presence/absence of bound 19A11, were also identical during the SMD runs. Taken together, our simulations show that the formation of salt bridges between two 19A11 Fabs and their corresponding Ecads in a strand–swap dimer (i.e., sets 1 and 2 of the 2ab condition) stabilizes the β-strand and pocket and strengthens adhesion. In contrast, when a salt bridge is formed between 19A11 and only one Ecad in a strand–swap dimer, the β-strand and pocket region are less stable and adhesion is not strengthened.

### 19A11 strengthens Ecad interactions at the single-molecule level.

**Fig. 5.**
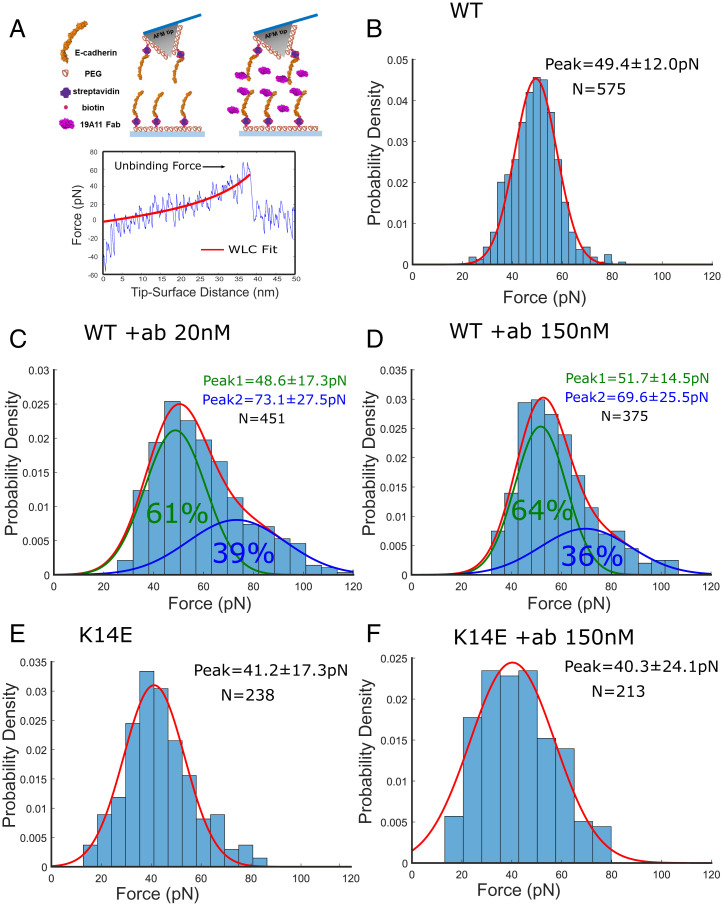
Direct, single-molecule measurements of 19A11-mediated strengthening of Ecad homophilic adhesion. (*A*) *Top Left:* Scheme for AFM experiment carried out in the absence of 19A11 (−ab). Ecads were immobilized on an AFM tip and substrate functionalized with PEG tethers. *Top Right:* Scheme for AFM experiment with antibody (+ab). Both the AFM tip and substrate were incubated with 19A11. *Bottom:* Example force curve. Stretching of the PEG tether, which served as a “signature” of a single-molecule unbinding event, was fit to a WLC model (red line). Experiments were performed with WT and K14E Ecad in the absence (−ab) and presence (+ab) of 19A11. Histograms of the unbinding forces were generated by binning the data in each condition using the Freedman–Diaconis rule. The optimal number of Gaussian distributions for each fit was determined using BIC. This analysis prescribed one Gaussian distribution for (*B*, *E*, and *F*) and two Gaussian distributions for (*C* and *D*). (*B*) Probability density of Ecad–Ecad unbinding forces measured in the absence of 19A11. Forces are Gaussian distributed (red line) with a peak force of 49.4 ± 12.0 pN. (*C*) Probability density of Ecad–Ecad unbinding forces in the presence of 20 nM 19A11 was best fit by a bimodal Gaussian distribution. While the first peak at 48.6 ± 17.3 pN (green line) corresponds to a “native” Ecad unbinding force, the second peak at 73.1 ± 27.5 pN (blue line) corresponds to strengthened adhesion. (*D*) Increasing the concentration of 19A11 in solution to 150 nM yields a similar bimodal Gaussian distribution with peaks at 51.7 ± 14.5 pN (green line) and 69.6 ± 25.5 pN (blue line). This demonstrates that the bimodal distribution of forces does not occur due to low 19A11-Ecad binding affinity but rather because 19A11 binds to Ecad in two distinct modes. (*E*) Probability density of Ecad K14E–K14E unbinding forces measured in the absence of 19A11. Forces are Gaussian distributed (red line) with a peak force of 41.2 ± 17.3pN. (*F*) Probability density of Ecad K14E–K14E unbinding forces measured in the presence of 150 nM 19A11. Since the K14E mutant abolishes the formation of the K14–D58 salt bridge, 19A11 Fab can no longer strengthen Ecad adhesion. Forces are single Gaussian distributed (red line) with a peak force of 40.3 ± 24.1pN.

To experimentally validate our simulations, we directly tested the effect of 19A11 binding on the strengthening of single Ecad interactions using AFM measurements. We immobilized the complete extracellular region of canine Ecad (EC1–5) on an AFM cantilever and glass substrate functionalized with polyethylene glycol (PEG) tethers, and measured Ecad–Ecad interactions in the presence and absence of 19A11 Fab (Fig. 5*A*). We have previously demonstrated that 19A11 Fab strengthens adhesion of Madin–Darby canine kidney cells expressing canine Ecad (9). Furthermore, the human Ecad and canine Ecad amino acid sequences are conserved with 91% sequence identity in the EC1 domain (*SI Appendix*, Fig. S4). However, since we performed AFM experiments with recombinant canine Ecad ectodomains, we independently verified 19A11 Fab binding using Western blotting (*SI Appendix*, Fig. S5).

A typical AFM measurement consisted of bringing the cantilever and substrate—both functionalized with Ecad—into contact and allowing the opposing cadherins to interact. The tip was then withdrawn from the substrate at a constant velocity, and the force required to rupture the Ecad–Ecad bond was measured. The interaction of opposing Ecads resulted in unbinding events that were characterized by the nonlinear stretching of PEG tethers, which were fit to a worm-like chain model (WLC) using least-squares fitting (Fig. 5*A*, *Bottom*).

Our AFM experiments directly tested Ecad binding strength under two conditions, with and without 19A11 Fab. AFM measurements without 19A11 Fab (“−ab”; Fig. 5*A*, *Top Left*) were performed to verify Ecad–Ecad binding and served as a control to benchmark 19A11 strengthening. Incubating both the coverslip and the cantilever with 19A11 and performing the AFM experiments in the presence of free 19A11 Fab in the measurement buffer constituted the “+ab” condition (Fig. 5*A*, *Top Right*). The −ab and +ab AFM experiments therefore mimic the 0ab and 2ab simulations.

To quantitatively compare binding strengths, we performed all AFM measurements at a single pulling velocity (1 µm/s). The resulting unbinding force distributions were fit to Gaussian distributions; the optimal number of Gaussian distributions to fit each dataset were informed by the Bayesian information criterion (BIC; *SI Appendix*, Fig. S6).

Unbinding forces between Ecad–Ecad bonds without the 19A11 bound (−ab) were best fit to a single Gaussian distribution and yielded an unbinding force of 49.4 ± 12.0 pN (Fig. 5*B*). However, when the experiments were performed in the presence of 20 nM 19A11, the unbinding force distribution was bimodal with one peak corresponding to strengthened Ecad–Ecad bonds (73.1 ± 27.5 pN; Fig. 5*C*) and one that was comparable to the −ab conditions (48.6 ± 17.3 pN; Fig. 5*C*). As predicted by the MD and SMD simulations, only ∼40% of Ecad interactions were strengthened (blue peak; Fig. 5*C*).

To confirm that the bimodal distribution of unbinding forces was due to 19A11 binding in two distinct modes (as predicted by the simulations), and not due to low affinity of Ecad for the mAb, we increased the concentration of the 19A11 Fab to 150 nM in the +ab experiments. Again, we measured a bimodal unbinding force distribution, but the unbinding forces still showed only ∼40% of Ecads that were strengthened (peak forces = 51.7 ± 14.5 pN and 69.6 ± 25.5 pN; Fig. 5*D*). Thus, in excellent agreement with the simulations, our AFM results showed that the binding of two 19A11 Fabs strengthened ∼40% of Ecad interactions.

To experimentally validate the obligatory role of salt bridge formation in strengthening adhesion, we performed similar AFM experiments with the Ecad K14E mutant, which abolishes the formation of one of the key salt bridges (K14–D58) between Ecad and 19A11. Previous structural studies (19) show that similar to wild-type (WT) Ecad, the K14E mutant interacts via strand–swap dimerization. Recent size–exclusion chromatography studies have shown that similar concentrations of 19A11 bind to both K14E mutant and WT Ecad (20). However, 19A11 does not recognize denatured Ecad in Western blots (*SI Appendix*, Fig. S5), suggesting that aside from the K14E-D58 salt bridge, other interactions also stabilize the antibody–Ecad complex. Our single-molecule AFM measurements show that in the absence of 19A11, the K14E unbinding force histogram was best fit to a single Gaussian distribution with a peak force of 41.2 ± 17.3 pN (Fig. 5*E*). Similarly, in the presence of 150 nM 19A11, the unbinding force histogram continued to be best described by a single Gaussian distribution with a peak force of 40.3 ± 24.1 pN (Fig. 5*F*), similar to what we observed in the absence of 19A11. These measurements confirm the key role played by the K14–D58 salt bridge on Ecad adhesion strengthening.

## Discussion

It has previously been shown that a key factor for Ecad strand–swap dimer formation is the stabilization of the swapped β-strand (residues 1–12) (16). Previous studies also show that residues 22 to 28 within the hydrophobic pocket enhance Ecad *trans* dimerization affinity by forming hydrogen bonds, specifically Asp1–Asn27 and Val3–Lys25, with the β-strand of its partner Ecad (17). Our X-ray crystal structure demonstrates that 19A11 mAb binds to the EC1 domain between the β-strand and the pocket region of Ecad. The binding interface observed in the crystal structure corresponds to previous epitope maps for 19A11 binding to Ecad (9). Several residues that were previously determined to be important for 19A11 recognition, such as R70 and P16, are present in this interface (9). Notably, the Ecad–19A11 Fab complex was copurified by size exclusion chromatography prior to cocrystallization trials and no significant lattice contacts are formed between 19A11 and Ecad, other than the paratope–epitope interactions, as described in this study.

Using MD simulations and AFM force measurements, we show that 19A11 forms two key salt bridge interactions with Ecad that stabilize both the swapped β-strand and the pocket region, which houses a W2 from its binding partner. Consequently, to strengthen Ecad adhesion, one of these salt bridges needs to be formed between both Ecads in the *trans* dimer and their bound 19A11. Abolishing one of these salt bridges (using the K14E mutant) eliminates adhesion strengthening. Due to the stochastic formation of salt bridges, 19A11 interacts with Ecad in two distinct modes: one that strengthens the Ecad–Ecad bond and one that does not change its adhesion.

In addition to forming robust strand–swap dimers, Ecads also adhere in a weaker X-dimer conformation, mediated by a salt bridge between K14 and D138 on the opposing Ecads (19, 21). X-dimers are believed to serve as intermediates in the pathway to strand–swap dimer formation (19, 22–24) and dissociation on the cell surface (25). Since residue D58 on the 19A11 heavy chain forms a salt bridge with K14 on Ecad, it is likely that 19A11 binding blocks X-dimer formation. In agreement with this suggestion, a recent cryogenic electron microscopy study shows that 19A11 selectively interacts with Ecad strand–swap dimers and does not bind to X-dimers (20). While blocking X-dimer formation could potentially prevent strand–swap dimer dissociation and thus strengthen adhesion, our data suggest that the force-induced dissociation of strand–swap dimers do not involve an X-dimer intermediate. Our AFM experiments demonstrate that the K14E mutant, where X-dimer formation is blocked, has an unbinding force similar to that of WT Ecad (Fig. 5 *B* and *E*). Similarly, our SMD simulations show that Ecad strand–swap dimers do not adopt an X-dimer conformation during dissociation. Instead, our data demonstrate that salt bridge–mediated stabilization of both the swapped β-strand and the pocket region are the dominant mechanism by which 19A11 strengthens Ecad adhesion. However, our study does not address whether X-dimers play a role in the force-free dissociation of the 19A11–Ecad complex or examine the role of X-dimers in the dissociation of the 19A11–Ecad complex on the cell surface. Furthermore, a recent study shows that Ecad bound to 19A11 adopts a twisted conformation which may potentially impact the stability of strand–swap dimers (20). Finally, in addition to binding in *trans* conformations, neighboring Ecads on the same cell surface also form *cis* dimers (26). However, 19A11 binding does not block the *cis* dimer interface and consequently is unlikely to interfere with *cis* dimer formation.

We note that due to the large size of our simulation system (∼880,000 atoms for Ecad bound to 19A11), the force used to unbind the protein in the constant force SMD simulations is relatively high. Therefore, in addition to constant force SMD simulations, we also performed SMD simulations at a constant pulling velocity (5 nm/ns; *SI Appendix*, Fig. S7). While the constant pulling velocity SMD simulations mimic the AFM experiments in silico, the unbinding forces in the AFM experiments and SMD simulations cannot be directly compared because of their different pulling velocities. However, the maximum forces measured in the simulations serve as a qualitative benchmark of the strength of the Ecad *trans* dimer. As anticipated from the AFM experiments, our constant-velocity SMD simulation data showed that the average maximum forces observed in the 0ab, 1ab, and weaker 2ab (sets 3–5) conditions were comparable. In contrast, the stronger 2ab (sets 1, 2) conditions had a higher average maximum force (*SI Appendix*, Fig. S7).

We have previously shown that 19A11 binding to Ecad ectodomains in Colo 205 cells induces p120-catenin dephosphorylation, which correlates with adhesion activation (9). While the current study does not investigate these intracellular consequences of 19A11 binding, we show that 19A11 also strengthens Ecad ectodomain adhesion, independent of the cytoplasmic region. Our work offers principles for the design of mAbs to enhance cadherin adhesion. Unlike integrin-activating antibodies that regulate ectodomain conformation (6), we show that 19A11 strengthens adhesion, not by inducing gross conformational changes in the Ecad ectodomain but rather by selectively stabilizing the swapped β-strand and its complementary binding pocket. Consequently, our results demonstrate that selectively targeting these structural and energetic determinants of strand–swap dimer formation is sufficient to strengthen cadherin adhesion.

## Materials and Methods

### Purification of human Ecad EC1–2 for X-ray crystallography.

We used residues 155 to 371 to encompass EC1–2 of the human Ecad extracellular domains; the signal sequence and prodomain were deleted (Δ1–154). EC1-2 was expressed as a fusion protein by attaching 6× His-tagged SMT3 to the N terminus (27). The EC1-2 construct was cloned into a pET His6 Sumo TEV LIC cloning vector (1S) (Addgene plasmid no. 29659) and expressed in Rosetta 2(DE3)pLysS *Escherichia coli* (Novagen 71401-3). Cultures were grown in autoinduction media (28) overnight and harvested via centrifugation. Thawed bacterial pellets were lysed by sonication in 200 mL buffer containing 25 mM Hepes pH 7.0, 500 mM NaCl, 5% glycerol, 0.5% CHAPS, 10 mM imidazole, 10 mM MgCl_2_, and 3 mM CaCl_2_. After sonication, the crude lysate was clarified with 2 µL (250 units/µL) benzonase and incubated while mixing at room temperature for 45 min. The lysate was then clarified by centrifugation at 10,000 rev min^−1^ for 1 h using a Sorvall centrifuge (Thermo Fisher Scientific) followed by filtration via 0.45 µm syringe filters. The clarified supernatant was then passed over an Ni-NTA His-Trap FF 5 mL column (GE Healthcare), which was pre-equilibrated with loading buffer composed of 25 mM Hepes pH 7.0, 500 mM NaCl, 5% glycerol, 20 mM imidazole, and 3 mM CaCl_2_. The column was washed with 20 column volumes (CV) of loading buffer and was eluted with loading buffer plus 500 mM imidazole in a linear gradient over 10 CV. Peak fractions, as determined by ultraviolet absorbtion at 280 nm, were pooled and concentrated to 10 mL. Pooled fractions were dialyzed overnight against 4 L buffer containing 500 mM NaCl, 25 mM Hepes, 5% glycerol, and 3 mM CaCl_2_ (SEC buffer) with His-tagged Ulp1 protease added to cleave the 6× His-SMT3 fusion protein at a ratio of 1 mg Ulp1 for 1,000 mg protein. Dialysate was passed over an Ni-NTA His-Trap FF 5 mL column to remove 6× His-SMT3 fusion protein and Ulp1. Flow-through from the Ni column was concentrated to 5 mL and passed over a Superdex 75 size exclusion chromatography column (GE) equilibrated with SEC buffer. The peak fractions were collected and analyzed for the presence of the protein of interest using Sodium dodecyl-sulfate polyacrylamide gel electrophoresis (PAGE). The peak fractions were pooled and concentrated using Macrosep 20 mL 10K MWCO protein concentrators (Pall). Aliquots were flash-frozen in liquid nitrogen and stored at −80 °C until use for the preparation of the Ecad EC1-2 Fab complexes.

### Purification of 19A11 Fab and Fab–Ecad complexes for X-ray crystallography.

Sequence coding for the heavy chain of the 19A11 Fab fragment was cloned into pcDNA3.4 with a Twin-Strep tag added after the C-terminal residue (SAWSHPQFEKGGGSGGGSGGGAWSHPQFEK*). ExpiCHO cells (Thermo Fisher) were transfected with the appropriate light chain and heavy chain–encoding plasmids for each Fab following the ExpiFectamine CHO Transfection Kit (Thermo Fisher) high-titer protocol. To obtain a single pure species of 19A11 for crystallography, a minor glycosylated product (10% of the total) was removed by incubating with ConA slurry (GE Healthcare) for 4 h at 4 °C on a rotator. Purification of StrepTag Fabs from ExpiCHO culture medium was performed using StrepTactin XT Superflow High Capacity resin (IBA) and elution with 50 mM biotin, followed by buffer exchange with PD-10 columns to 50 mM Tris pH 8.0, 150 mM NaCl, and 3 mM CaCl_2_. Isolation of a single species for each Fab was verified by PAGE, and activation of cellular Ecad was confirmed by a Colo 205 activation assay (9). The Ecad was incubated with a 1.6× molar excess of ConA-purified 19A11–Fab and incubated overnight at 4 °C. The complex was purified via size exclusion chromatography with a Superose 6 10/300 GL column and concentrated to 10.3 mg/mL in 50 mM Tris, 150 mM NaCl, and 3 mM CaCl_2_, pH 8.0.

### Crystallization of human Ecad with 19A11 Fab.

Protein complex (0.1 µL) was mixed with 0.1 µL crystallization solution (Wizard 3/4, well H12, Rigagku Reagents) containing 15% (wt/vol) PEG-20000, 100 mM Hepes/NaOH (pH 7.0), and equilibrated against 50 µL crystallization solution in an MRC2 vapor diffusion tray (SWISSCI). Crystals were harvested and cryoprotected with crystallization solution supplemented with 20% ethylene glycol and flash-frozen in liquid nitrogen.

### Data collection and structure solution.

Diffraction data was collected at the Life Sciences Collaborative Access Team beamline 21-ID-F at the Advanced Photon Source (Argonne National Laboratory) on a Rayonix MX300 CCD detector at a wavelength of 0.97872Å. Data were indexed and integrated with XDS and scaled with XSCALE (29). The structure was solved with Phaser (30) using PDB accession code 2O72 as a search model for Ecad and PDB accession code 4WEB as a search model for 19A11. The model was refined with iterative rounds of refinement with Phenix (31) and manual model building in Coot (32, 33). The quality of the structure was checked with Molprobity (34).

### Purification of canine WT Ecad and K14E Ecad mutant ectodomains for AFM.

The generation of WT Ecad and K14E mutant monomer plasmids containing a C-terminal Avi tag has been described previously (35, 36). The plasmids were incorporated into pcDNA3.1(+) vectors and were transiently transfected into HEK 293T cells using PEI (Milipore Sigma) as previously described (37). Three days posttransfection, conditioned media were collected for protein purification. Purification of WT Ecad and K14E mutants were performed using methods described previously (24, 35, 38). Media containing His-tagged Ecads were passed through a chromatography column containing Ni-NTA agarose beads (Qiagen). Beads were then washed with a pH 7.5 biotinylation buffer (25 mM Hepes, 5 mM NaCl, and 1 mM CaCl_2_). Ecads bound to the Ni-NTA beads were biotinylated with BirA enzyme (BirA 500 kit; Avidity) for 1 h at 30 °C. Following biotinylation, free biotins were removed using the Ni-NTA column and biotinylated Ecads bound to Ni-NTA beads were eluted using a pH 7.5 buffer containing 200 mM imidazole, 20 mM Na_2_HPO_4_, 500 mM NaCl, and 1 mM CaCl_2_.

### Single-molecule AFM experiments.

Purified canine Ecad monomers were immobilized on AFM cantilevers (Hydra 2R-50N; AppNano) and glass coverslips (CS) as described previously (38, 39). Briefly, the CS and cantilevers were cleaned with 25% H_2_O_2_/75% H_2_SO_4_ overnight and washed with Milli-Q water. The CS were then cleaned with 1 M KOH and washed with Milli-Q water. Both the CS and cantilevers were washed with acetone and functionalized using 2% (vol/vol) 3-aminopropyltriethoxysilane (Millipore Sigma) solution dissolved in acetone. *N*-hydroxysuccinimide ester functionalized PEG spacers (MW 5000, Laysan Bio) were covalently attached to the silanized AFM tip and coverslip (100 mg/mL in 100 mM NaHCO_3_ dissolved in 600 mM K_2_SO_4_, for 4 h); 10% of the PEG spacers were decorated with biotin groups. Prior to a measurement, the functionalized AFM cantilever and coverslip were incubated overnight with BSA (1 mg/mL) to further reduce nonspecific binding. The tip and surface were then incubated with streptavidin (0.1 mg/mL for 30 min; Thermo Fisher) and biotinylated canine Ecad (200 nM for 1 h) was attached to the streptavidin. Finally, the surfaces were incubated with 0.02 mg/mL biotin for 10 min to block the free biotin binding sites on streptavidin.

Force measurements were performed using an Agilent 5500 AFM with a closed-loop scanner. The force measurements were performed in a pH 7.5 buffer containing 10 mM Tris⋅HCl, 100 mM NaCl, 10 mM KCl, and 2.5 mM CaCl_2_. Cantilever spring constants were measured using the thermal fluctuation method (40). Unbinding events, which were characterized by stretching of the PEG tethers, were fit to a WLC model using a least-squares fitting protocol and specific events were chosen by discarding events that had an rms error (rmse) greater than the mean plus 1 SD of all rmses. In addition, since full-length PEG has a contour length of ∼30 nm, events with contour lengths less than 30 nm were excluded. Furthermore, persistence lengths were constrained between 0.1 nm and 1 nm.

Histograms of unbinding forces were generated by binning the data for each experimental condition using the Freedman–Diaconis rule. The BIC was used to determine the optimal number of Gaussian subpopulations for each dataset and avoid overfitting.

### MD simulations and analysis.

MD simulations were performed with GROMACS 2020.1 using the FARM high-performance computing cluster at University of California, Davis as described previously (38). Simulations were performed using OPLS-AA/L force field (41) and TIP4P water models. A radius cutoff of 10Å was used for Van der Waals and electrostatic interactions. Electrostatic energy was computed using the particle mesh Ewald method with a 0.16-grid spacing for fast Fourier transform. The Ecad crystal structures in the absence of 19A11 (PDB accession code 2O72) and in the presence of 19A11 (PDB accession code 6CXY) were equilibrated by performing 60-ns MD simulations. The protein structure was placed in the center of a dodecahedral box such that no atom of the protein was closer than 1 nm to any boundary throughout the duration of the simulation. The box was solvated by adding water molecules and charge-neutralized by adding ions (150 mM NaCl, 4 mM KCl, and 2 mM CaCl_2_). Each simulation system contained ∼92,500 atoms. Each system was relaxed using energy minimization and stabilized by equilibration under isothermal–isochoric and isothermal–isobaric conditions, using a modified Berendsen thermostat and Berendsen barostat. Following equilibration, a 60-ns MD simulation was performed with 2-fs integration steps. The structures equilibrated after ∼20 ns. Equilibration was monitored by calculating the backbone RMSD of the structures relative to the initial structure. The C-α RMSF of each residue in the Ecad EC1 domain (residues 1–100) during the final 10 ns MD was calculated using the *gmx rmsf* module. The distances between charged atoms for the salt bridges E13–R99 and K14–D58 were calculated using the *gmx pairdist* module.

### Constant-force/constant-velocity SMD simulations and analysis.

The last frame of each MD simulation was placed in the center of a rectangular box such that interacting Ecads were parallel with the longest axis of the box and no atom of the protein was closer than 1 nm to any boundary (30 × 12 × 8 nm for the 0ab conditions; 30 × 15 × 15 nm for the 1ab/2ab conditions). Each simulation system contained ∼380,000 atoms for the 0ab conditions and ∼880,000 atoms for the 1ab/2ab conditions. The simulation system was relaxed and equilibrated using the same methods as the MD simulations without the isothermal–isochoric condition. During the SMD, the C terminus of one Ecad was fixed while the other Ecad was pulled on a group of residues near the C terminus of the second domain (residues 151–166, 174–186, and 208–213) in a direction aligned with the longest axis of the box. The constant-force SMD simulations were performed at a pulling force of ∼665 pN (400 kJ⋅mol^−1^⋅nm^−1^) and the constant-velocity SMD simulations used a pulling rate of 5 nm/ns and a spring constant of 400 kJ·mol^−1^·nm^−2^.

The change in the Ecad interfacial area was estimated from the ΔSASA (ΔSASA = SASA [protein A] + SASA [protein B] − SASA [protein A + protein B]), which was calculated using the *gmx sasa* module. The distance between the center of mass of W2 and the corresponding pocket (residues 22–28, 36, 78–80, and 89–92) was obtained using the *gmx pairdist* module.

## Supplementary Material

Supplementary File

Supplementary File

## Data Availability

The X-ray crystallographic structure of Ecad EC1–2 bound to 19A11 is available in the Protein Data Bank with accession code 6CXY (42). AFM and MD simulation data are made available in the *SI Appendix*. All study data are included in the article and/or *SI Appendix*.
