# Supplementary material for: Molecular mechanism for strengthening E-cadherin adhesion using a monoclonal antibody
Source: Proc Natl Acad Sci U S A. 2022 Aug 3;119(32):e2204473119. doi: 10.1073/pnas.2204473119 (PMC9371698; doi:10.1073/pnas.2204473119)
Supplement: Supplementary File [file pnas.2204473119.sapp.pdf]

Supplementary Information

Title: Molecular mechanism for strengthening E-cadherin adhesion using a monoclonal antibody

Authors: Bin Xie [1, 2], Allison Maker [3, 4], Andrew V. Priest [2], David M. Dranow [5, 6], Jenny N. Phan [5, 6], Thomas E. Edwards [5, 6], Bart Staker [5,7,] Peter J Myler [5,7,8,] Barry M. Gumbiner [3, 4, 8], Sanjeevi Sivasankar [1, 2, *]

Affiliations: [1] Biophysics Graduate Group, University of California, Davis, CA; [2] Department of Biomedical Engineering, University of California, Davis, CA; [3] Seattle Children's Research Institute, Center for Developmental Biology and Regenerative Medicine, Seattle, WA; [4] Department of Biochemistry, University of Washington, Seattle, WA; [5] Seattle Structural Genomics Center for Infectious Disease (SSGCID), Seattle, WA; [6] UCB Pharma, Bainbridge Island, WA; [7] Center for Global Infectious Disease Research, Seattle Children's Research Institute, Seattle, WA; [8] Department of Pediatrics, University of Washington, Seattle, WA.

***Corresponding Author:** ssivasankar@ucdavis.edu

Table S1. Data collection and refinement statistics

Beamline	APS 21-ID-F
Space group	C2
Cell dimensions	
a, b, c (Å)	122.68, 77.47, 110.94
α, β, γ (°)	90.000, 92.905, 90.000
Resolution (Å)	50.0–2.20 (2.26–2.20)
No. reflections	221,388 (16,425)
No. unique reflections	52,838 (3,868)
Rmeas	0.094 (0.672)
Rmerge	0.082 (0.587)
I/σ(I)	13.40 (2.60)
CC1/2 (%)	99.7 (78.4)
Completeness (%)	99.9 (99.9)
Redundancy	4.2 (4.2)
Refinement	
Resolution (Å)	40.09–2.20
No. reflections	52,833
Rwork / Rfree	0.1626 / 0.1970 (0.2370 / 0.3102)
No. atoms	
Protein	4,849
Ligand/ion	90
Water	646
B factors	
Protein	35.04
Ligand/ion	56.07
Water	43.29
R.M.S. deviations	
Bond lengths (Å)	0.007
Bond angles (°)	0.892
Ramachandran	
Preferred (%)	97.91
Allowed (%)	1.93
Outliers (%)	0.16

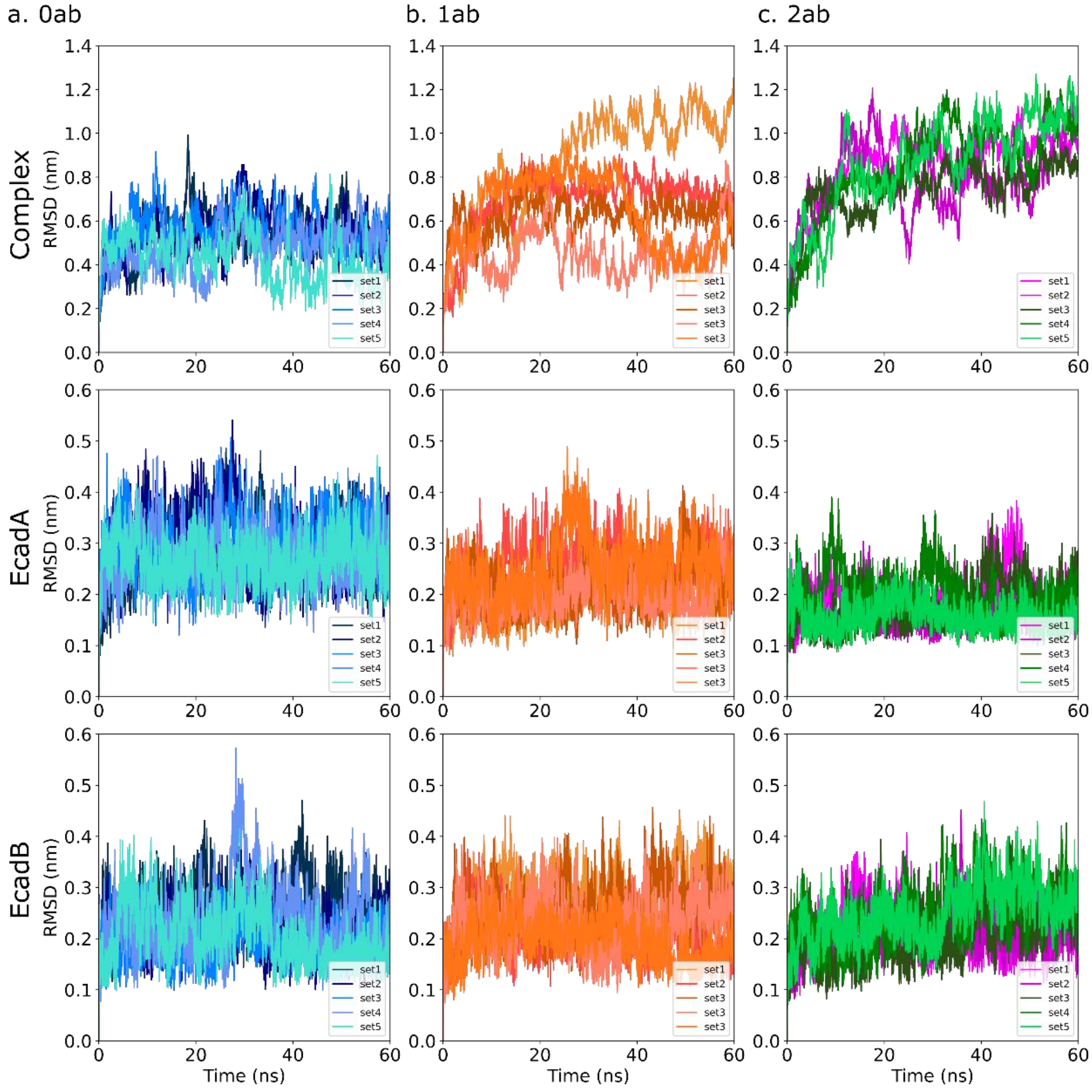

Fig. S1. **Protein backbone RMSD of every MD simulation frame relative to the structures at the start of the simulation.** RMSD values observed for the overall complex (top), and for EcadA/EcadB monomer (middle/bottom) in the (a) 0ab condition, (b) 1ab condition, and (c) 2ab condition. RMSD values stabilize after 25ns for all simulations, suggesting the structures are well equilibrated.

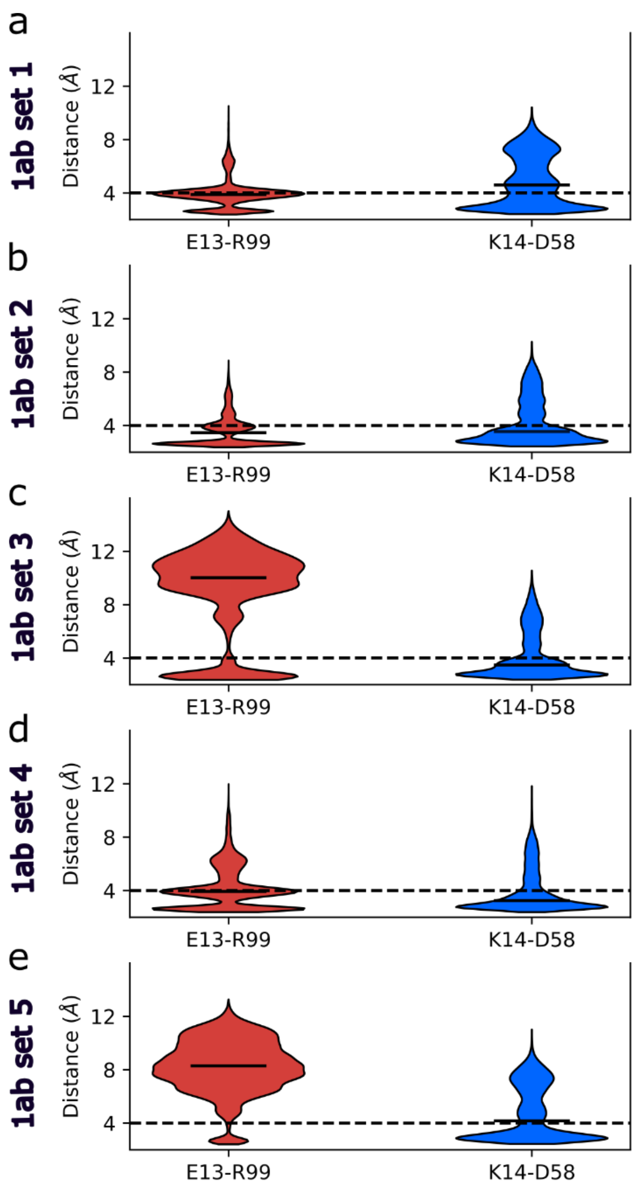

Fig. S2. Formation of the E13-R99 and/or K14-D58 salt bridges during the 1ab MD simulation. Violin plots of the distance between charged atoms in the E13-R99 and K14-D58 salt bridges during the last 40 ns of each MD simulation: (a) set 1, (b) set 2, (c) set 3, (d) set 4, (e) set 5. All of the simulation sets have at least one salt bridge formed during the MD.

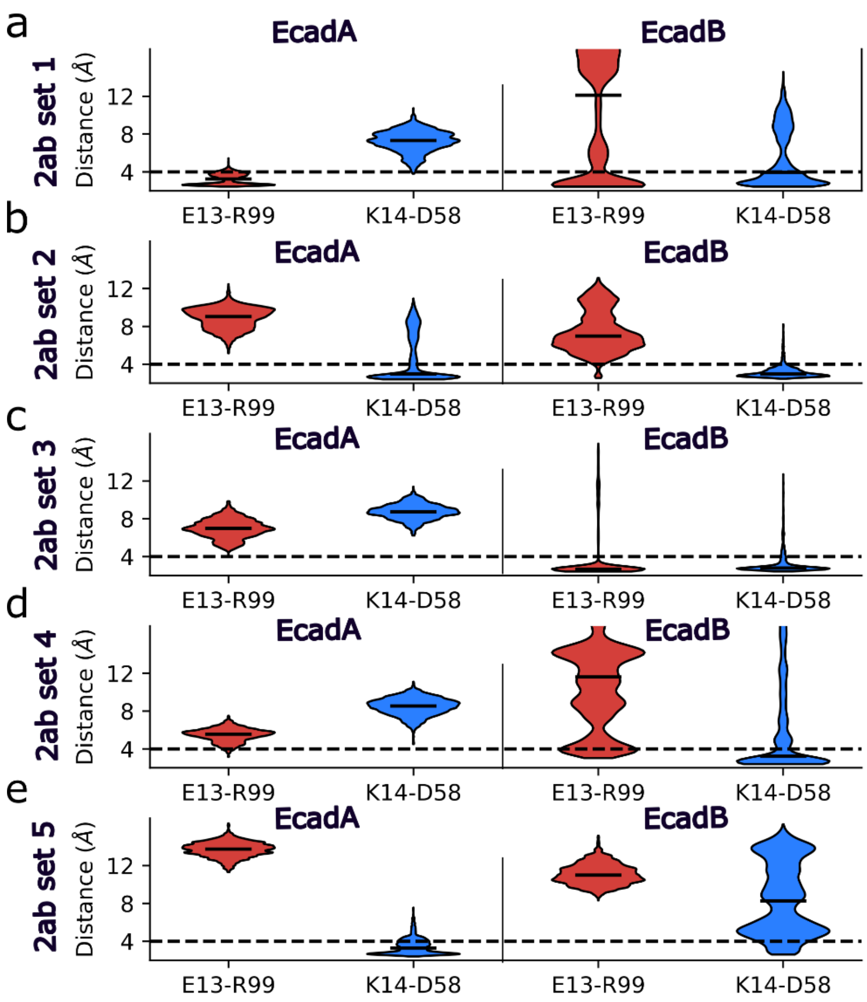

Fig. S3. Analysis of the E13-R99 and/or K14-D58 salt bridge during the constant force SMD simulation. Violin plots of the distance between charged atoms in the E13-R99 and K14-D58 salt bridges observed throughout each SMD simulation: (a) set 1, (b) set 2, (c) set 3 (d) set 4, and (e) set 5. In set 1 and 2, at least one salt bridge was formed on each Ecad while for set 3, 4 and 5, only one Ecad formed a salt bridge. Almost all of the salt bridges that were formed during the MD simulation were retained during the SMD simulation.

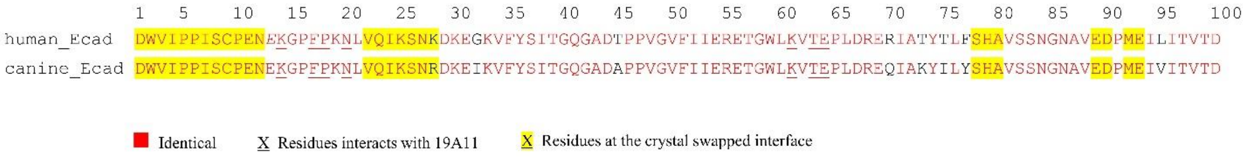

Fig. S4. Sequence comparison between human Ecad and canine Ecad shows 91% sequence identity.

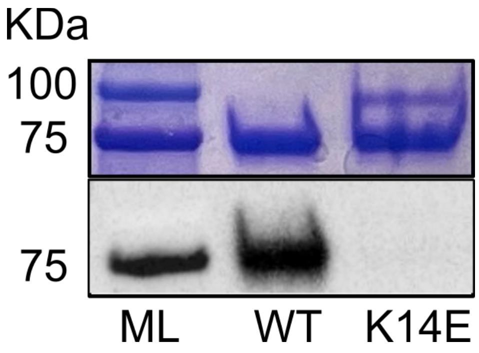

Fig. S5. SDS-PAGE gel and Western-blot of both canine wild-type (WT) Ecad and canine K14E Ecad. Molecular ladder (M) with molecular weight 75KDa and 100KDa is shown on the SDS-PAGE gel, and molecular weight 75KDa is shown on the western blot. Western blotting was performed using 19A11 Fab as the primary antibody with horseradish peroxidase conjugated secondary antibody. Protein samples were detected with a WesternBright Chemiluminescence Kit (K-12045; Advansta).

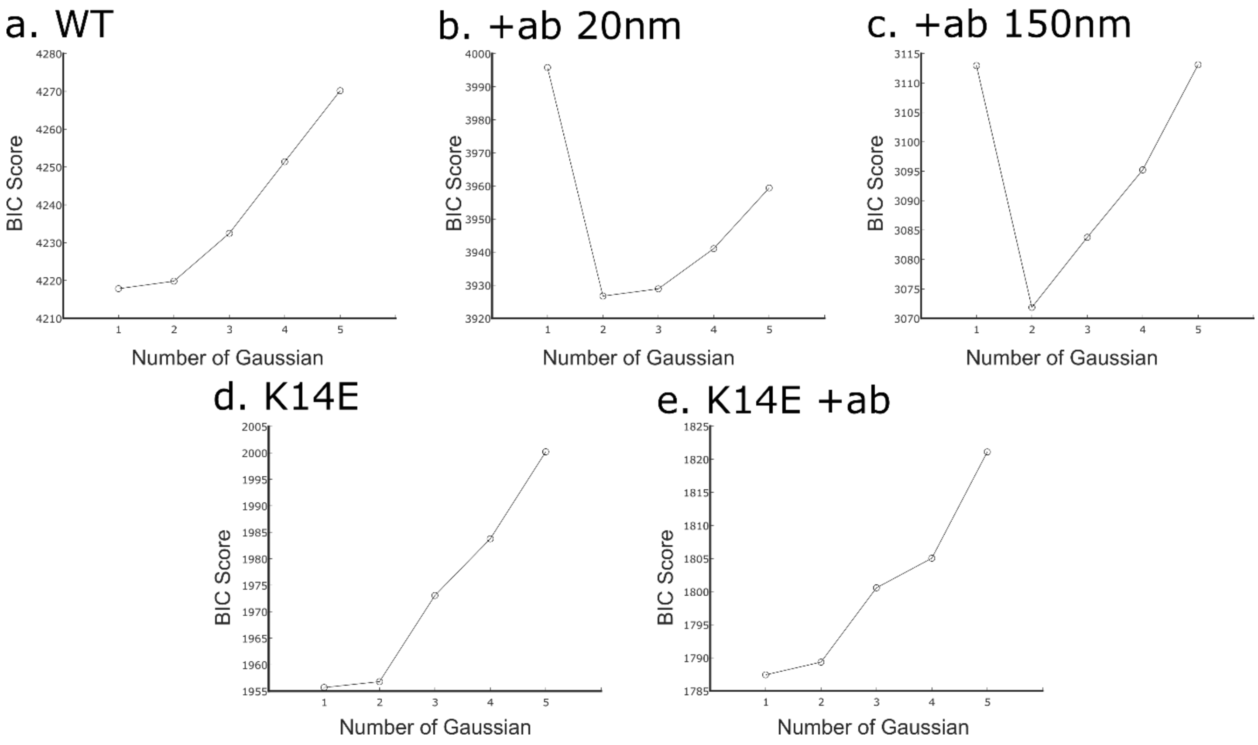

Fig. S6. **Bayesian information criterion (BIC) predicts the optimal number of Gaussians in the unbinding AFM force histograms.** BIC ascribes a penalty to reflect the fact that more free parameters will always yield a better fit; lower BIC scores corresponds to more likely model. A single Gaussian distribution optimally describes (a) WT-Ecad, (d) K14E Ecad, and (e) K14E +ab. A bimodal Gaussian distribution best describes (b) +ab 20nm and (c) +ab 150nm, force distribution.

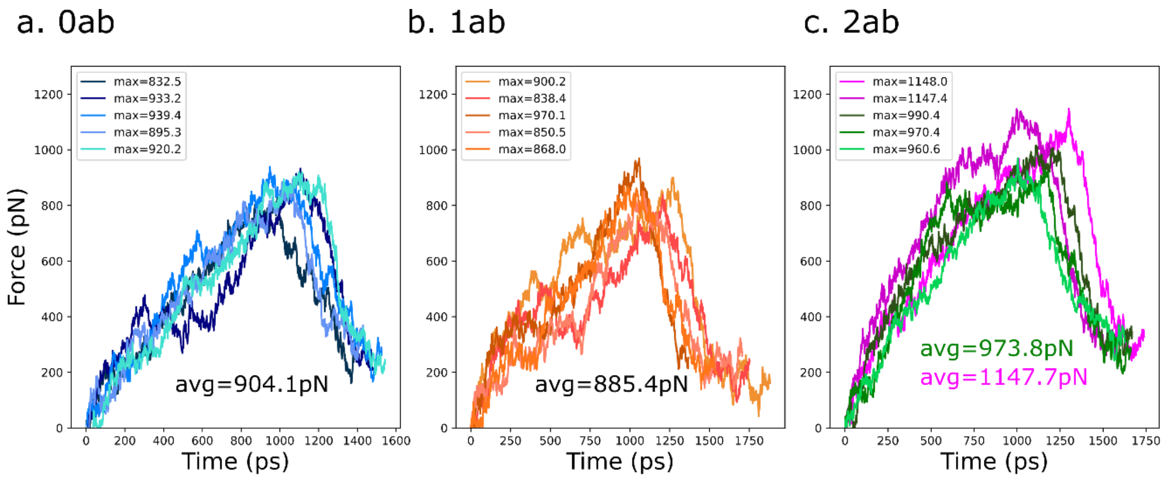

Fig. S7. Forces recorded during constant velocity SMD simulations. (a) 0ab condition, (b) 1ab condition, and (c) 2ab condition. The average maximum forces measured in the 0ab and 1 ab condition are similar with values of 904.1 pN and 885.4 pN respectively. However, there are two populations observed in the 2ab conditions: set 1/set 2 have a significantly higher average maximum force of 1147.7pN, while set 3/set 4/ set 5 are weaker with an average maximum forces of 973.8pN.

Supplemental Movie 1. Example constant-force SMD simulations. One constant-force SMD example from each condition: 0ab (set 1), 1ab (set 4), 2ab weak conformation (set 5), and 2ab strong conformation (set 2) are shown. Ecad strand-swap dimers in the 0ab, 1ab and 2ab weak conformation break significantly faster compared with the 2ab strong conformation. Color scheme: Ecad (cyan and green), 19A11 heavy chain (magenta), and 19A11 light chain (orange).

Supplemental Methods

SDS-PAGE Gel and Western blots

Purified canine Ecad and K14E Ecad mutants were prepared in 1× sodium dodecyl sulfate (SDS) sample buffer (10 mM Tris, pH 6.8, 1% SDS, 10% glycerol, 0.005% bromophenol blue, and 2% β-mercaptoethanol). Samples were heated in 95ºC water for 10mins before loading into the SDS-PAGE gel (BIO-RAD, Mini-PROTEAN TGX Precast Gels). Protein Molecular ladder (BIO-RAD, Precision Plus Protein Duel Color Standards) was also loaded into the gel for comparison. A running buffer containing 25mM Tris, 192mM glycine and 0.1% (w/v) SDS, pH 8.3 was used. Electrophoresis were performed at constant voltage of 200 V for 30 mins. Samples were visualized by first staining in Coomassie blue staining buffer and then destained in buffer containing 7% acetate acid, 20% ethanol and 73% DI-water.

Western blots were performed by running SDS-PAGE gel (as described above) without staining the gel. Mouse 19A11 Fab, diluted in PBST (phosphate-buffered saline with 0.1% Tween 20), was used as the primary antibody against canine WT Ecad and K14E Ecad mutants. HRP (Horseradish peroxidase) conjugated secondary antibody against mouse was used in 1:1000 dilution in PBST. Protein samples were detected with a WesternBright Chemiluminescence Kit (K-12045; Advansta). Images were acquired using Image Lab software from Bio-Rad.
